# Temperature driven hibernation site use in the Western barbastelle *Barbastella barbastellus* (Schreber, 1774)

**DOI:** 10.1038/s41598-020-80720-4

**Published:** 2021-01-14

**Authors:** Luc De Bruyn, Ralf Gyselings, Lucinda Kirkpatrick, Alek Rachwald, Grzegorz Apoznański, Tomasz Kokurewicz

**Affiliations:** 1 Research Institute for Nature and Forest (INBO), Brussels, Belgium; 2 Evolutionary Ecology Group, University of Antwerp, Antwerp, Belgium; 3 Forest Ecology Department, Forest Research Institute, Sękocin Stary, Raszyn, Poland; 4 Department of Vertebrate Ecology and Paleontology, Institute of Environmental Biology, Wrocław University of Environmental and Life Sciences, Wrocław, Poland

**Keywords:** Ecology, Zoology

## Abstract

In temperate regions, winter is characterized by cold temperatures and low food availability. Heterothermic animals can bridge this period by entering a state of torpor characterized by decreased body temperature and reduced metabolic rate. Hibernation site choice is crucial since temperature conditions in the hibernaculum will impact torpor. We analysed temperature-dependent hibernation site use of *Barbastella barbastellus*. Bats and temperature were monitored in an underground system (1999–2019) and standalone bunkers (2007–2019) in Western Poland. During the winter of 2017–2018 we analysed the thermal variability of the hibernacula. Seasonal variation is higher in bunkers and thus temperatures get colder in winter than in the underground system. On the other hand, short-term variability (thermal variability index) in the bunkers was lower than in the underground system. This makes bunkers a more stable environment to hibernate for cold dwelling bats in warm winters, when temperatures in the bunkers do not get below freezing. Bats use both the warm underground system and the colder bunkers. During the last decade, a continuous series of warm winters occurred and the population of barbastelle bats partly moved from the underground system to the bunkers. These present temperature increases broadened the range of potential hibernation sites for barbastelles. Our study indicates that long-term trends, seasonal variation and short-term variability in temperatures are all important and should be analysed to investigate hibernaculum use by bats. Our study shows that small hibernation sites may become more important in the future.

## Introduction

During winter, when ambient temperatures are low, many animals face a high energy need combined with a lack of food. To cope with this period of limited energy supply, during which the cost may prohibit homeothermy, heterotherms enter a state of torpor, characterized by a decrease in their body temperature set point and a drastically reduced metabolic rate^1–6^. For example, the torpor metabolic rate for *Myotis lucifugus* at 2 °C body temperature is only 1% of the resting metabolic rate at a body temperature of 37 °C^5^. Using prolonged periods of torpor, hibernators can survive on stored fat for months^1^. However, surviving the whole winter season on fat reserves can be a challenge, especially when harsh conditions occur at the end of the winter^7,8^.

In torpor, heterotherms can lower their body temperature down to a minimum temperature (T_min_) at which the rate of energy expenditure is minimal. At ambient temperatures below T_min_ the rate of energy expenditure increases sharply. The minimum body temperature T_min_ is species dependent^9^. Traditionally it was thought that heterotherms should select microclimates with a temperature close to T_min_ to minimize their metabolic rate and energy consumption^10–12^, although the reported range of temperatures at which heterotherms of different species were found hibernating is very wide and there is a considerable overlap in the reported range of temperatures for different species^13–19^. Today it is well understood that there are also costs associated with lowering the body temperature in torpor^20^. Several negative physiological impacts have been reported, including reduced immunocompetence^21,22^, sleep deprivation^23^ and reduced protein synthesis^24^. Furthermore, torpor can reduce a hibernator’s ability to escape from predators, or can lead to missed opportunities such as mating^20^. Microclimate selection therefore is a process of balancing the trade-off between maximizing energy conservation and minimizing the negative consequences of torpor, as was elaborated extensively in an optimal hibernation theory framework by Boyles, et al.^20^. Optimal microclimate selection can vary not only between species, but also between juveniles and adults^25,26^, between sexes, or even between individuals^26,27^. Optimal microclimate selection will also change during the course of the winter, with bats often switching between microhabitats^10,28–31^, indicating that bats optimize their hibernation strategy by using both physiological and behavioral mechanisms^20,27^.

The choice of an optimal hibernation site is constrained by the availability of hibernacula, and subsequently the microsites within said hibernacula in the landscape, while the availability of microsites within a hibernation site are determined by the physical laws of heat transport. In a cave environment, sites deep in the cave will have on average higher and more stable temperatures. Closer to the entrance, temperatures will drop to lower average values when the winter progresses, but they will be more variable and thus less predictable^10,19,29,32^. This unpredictability can make a choice for higher temperatures more optimal^33,34^. Boyles, et al.^32^ introduced the thermal variability index (TVI), which quantifies the temperature variation in a hibernaculum, and used this metric to compare variability between different microsites in a cave system. The TVI is in essence a modified form of standard deviation that estimates the magnitude of variation in ambient temperature over a given time period in relation to the ambient temperature at the beginning of the time period. Biologically this represents the variation away from the ambient temperature that a bat can expect when it chooses a microsite for hibernation^32^.

Since microclimate temperatures are ultimately driven by the outside temperature, climate change can have a significant effect on the availability and choice of hibernation sites. Most climate change impact studies focus on conditions during the summer or growing/reproduction season, despite winter conditions being more severely altered^35^. There is growing evidence that winter conditions are changing rapidly, resulting in an increasing average global temperature, a reduction in the number of frost days in temperate regions, shortening of the snow season and an increased rate of extreme warm temperature events^35–37^. Based on the size of the energy reserves at the onset of hibernation, the depletion rate during winter, and the length of the winter, Humphries, et al.^38^ developed a bioenergetic model that relates winter energetic requirements to ambient winter temperatures. The model suggests that only a relatively narrow combination of hibernaculum temperature and winter duration permits successful overwintering. For example, for *Myotis lucifugus*, their model indicates that for a winter length of 193 days, hibernation should take place between 0 and 12 °C, with a minimum energy consumption at 2 °C. Similar conclusions were obtained for *Myotis sodalis* albeit with a narrower temperature range^39^. This may render hibernating species particularly vulnerable to changing winter climatic conditions as ambient winter temperatures modify the microclimatic conditions within sites, potentially raising hibernacula temperatures above optimal hibernation temperatures and negatively impacting crucial life history phases such as hibernation^40^. Understanding the dynamics of microclimatic conditions in hibernation sites during the winter period is therefore critical to bat conservation^32,41^, particularly in the face of our changing climate. Therefore, a better understanding of hibernaculum use and site choice under different temperature conditions is necessary, with the monitoring of microclimatic conditions within winter sites of primary importance^32,41^.

Here, we focus on how bat species preferring cold temperatures may adjust hibernation site use, using the Barbastelle bat *Barbastella barbastellus* as an example. *Barbastella barbastellus* is a psychrophilic species reported to hibernate in exposed, dry (75–90% relative humidity) and well ventilated shelters with temperatures ranging between -3.0° and 6.5 °C, but usually above 0 °C^13,42^. In deep underground systems *B. barbastellus* is often found hibernating in the colder parts of the system near the entrance^15,16^. They also often use smaller shelters like cellars and bunkers^43,44^, and can even hibernate in trees^45^. *B. barbastellus* is among the seven European species most vulnerable for climate change effects^46^, it faces major reductions in its distribution range under many future climate change scenarios^40^, which are exacerbated by its sedentary nature^47^ (a study in Germany estimated the majority of movements as being between 4 and 44 km^48^). We studied numbers of *B. barbastellus* in a deep underground system, and in stand-alone above ground bunkers scattered in the vicinity of the underground system in Western Poland. Previous work by Gottfried, et al.^41^ found a decrease in the number of *B. barbastellus* hibernating in the underground system and an increase in the surrounding bunkers, but they did not find any significant correlation between the two. We also monitored the temperatures within the underground system and the bunkers during a full winter season (2017–2018) to analyse the thermal dynamics of both systems and microsite choice by *B. barbastellus*. In addition, we analysed changes in bat numbers in the underground system (1999–2019) and bunkers (2007–2009) and related these to temperature conditions to assess long term effects in hibernation site use.

In this study we want to assess the thermal characteristics of hibernation sites and relate this to hibernaculum use by the bats. We predict that thermal variability is higher in the above ground bunkers than in the underground system, and that temperatures in the bunkers drop to lower values as the winter proceeds. Since *B. barbastellus* is a psychrophilic species, we predict it will predominantly use the colder available microsites. We also predict that in warm winters the deep underground system becomes too warm for hibernating *B. barbastellus,* and they move to alternative hibernation sites.

## Materials and methods

### Fieldwork

Bats were monitored in an underground corridor system of the Natura 2000 site PLH080003 "Nietoperek" in Lubuskie Region, Western Poland (52°25′N, 15°32′ E) (Supplementary Figure S.1) and 19 bunkers in the neighbourhood of the underground system (Supplementary Figure S.2). The underground system and the bunkers are part of a military defence belt built in the period 1934–1944. In 1957 the military constructions were abandoned, and nature was left to take over the system. The fortifications consist of a large main underground corridor system (ca 32 km long, 20-30 m below ground) and one or two storey bunkers situated on the surface and partially destroyed by explosives (Supplementary Figure S.2).

Every year up to 40,000 bats of 13 species hibernate in both the main underground system "Nietoperek" and the surrounding bunkers, making it one of the largest and most important bat hibernation sites in Europe^49^. Bat populations in Nietoperek have been censused biennially between 1999 and 2005 and annually afterwards, making this an extremely valuable long-term database. For monitoring purposes the underground system is divided in 9 sections (143 subsections; Supplementary Figure S.1), each of which is counted by a single team of about 7 bat workers^49–51^. To ensure reliability of the data, teams were always led by a selected group of people that have a lot of experience with the system. To minimize disturbance, censuses in the main underground system are carried out in mid-January, the period when the number of hibernating bats is highest, during a single day from the sunrise to sunset. Annual monitoring of the bunkers started in 2007 and involves teams of 5–7 bat workers examining the bunkers the day after the visit to the underground system, counting the number of bats per room. Not all bunkers were counted every year, but all bunkers included in the dataset were counted at least six times during the study period. Bats were identified and counted visually, without handling the animals under the licences issued by the Regional Nature Conservation Management in Gorzów Wielkopolski.

### Temperature data collection

Long-term (1999–2019) temperature data consisting of three daily measurements (6am, 12 pm, 6 pm) were obtained from the nearest weather station of the National Institute of Meteorology and Water Management at Lubinicko-Świebodzin, 10 km from our study site. As a measure of winter climatic conditions, we calculated four parameters based on the temperature data for the period between 1 November and 15 January: the average daily mean temperature, the average daily minimum temperature, the number of frost days (total number of days when frost occurred at least part of the day, i.e. days with a minimum temperature below 0 °C) and the number of ice days (total number of days when frost occurred all day, i.e. days with a maximum temperature below 0 °C).

In the winter of 2017–2018, temperature loggers (iButtons models DS1921 and DS1925, Thermochron, Baulkham Hills, Australia) were deployed in 52 (sub)sections of the underground system and in 53 rooms of 7 bunkers. The corridors of the underground system in which loggers were placed consist of three types: a main corridor with a height of 3.7–4 m, large side corridors with a height of 3–3.5 m and small side corridors with a height of 2.2–2.5 m. If the height of the corridor was less than 3 m loggers were placed in the middle of the ceiling. In higher corridors loggers were placed at a height of 3–3.5 m, which corresponds to a height where most bats are found. At four control points, additional loggers were placed at lower heights, but they did not show any temperature difference compared with the loggers at the standard high height. In the bunkers loggers were always placed against the wall near the ceiling, which is 2–2.5 m high. Loggers recorded the temperature every 4 h with an accuracy of ± 0.5 °C. We used the temperature data from November to April to assess seasonal variation in the temperature of underground sections and bunker rooms with and without barbastelle bats.

### Data analysis

#### Climate parameter trends

Long-term (1999–2019) trends in the average daily mean temperature, the average daily minimum temperature, the number of frost days and the number of ice days were modelled with generalised linear models (GLM). We used a gaussian error distribution for the average daily mean temperature and the average daily minimum temperature, and a negative binomial distribution with a log link for the number of frost days and the number of ice days. All models were compared with an intercept only null model without a trend term. Model comparison was based on AIC values, with models with an AIC at least two units lower than the null model being considered significantly better.

#### Thermal variability index

To assess the short-term variability in temperatures at the 52 (sub)sections and 53 rooms with loggers, we calculated the Thermal Variability Index (TVI) as defined by Boyles, et al.^32^.

$$TVI_{x} = \sqrt {\frac{{\mathop \sum \nolimits_{i = 1}^{n} \left( {T_{0} - T_{i} } \right)^{2} }}{n - 1}}$$

where $x$ is the timeframe over which TVI is calculated, n is the number of times temperature is sampled across the calculation period, T_0_ is the temperature at the beginning of the timeframe and T_i_ is the i-th temperature measurement in the timeframe. TVI represents how much variation in temperature a bat will experience if it chooses a site at the beginning of the timeframe and stays there for the length of the timeframe. We used a timeframe of 14 days, as suggested by Boyles et al.^32^, to capture short-term temperature fluctuations. This period corresponds to an average torpor bout length of several species as found by Brack and Twente^52^. We chose January as a reference period because all censuses were carried out in this month and used each measuring time point in January as the starting point to calculate a TVI_14_ series for each site. The maximum of these values per time series was used as Thermal Variability Index for the site.

Thermal Variability Index was modelled against mean January temperature with a non-linear Generalised additive model (GAM)^53^, with object type (underground vs. bunkers) as factorial covariate and compared to a null model without object type as covariate. Model comparison was based on AIC values, with models with an AIC at least two units lower than the null model being considered significantly better.

#### Trends in bat numbers

To assess the trend in bat numbers in the underground system we summed the annual bat numbers counted in all 9 sections.

To assess the trend in bat numbers in the bunkers, we summed the annual bat numbers in all the bunkers after imputing missing values, since not all bunkers were examined every year. We used the multiple imputation method proposed by Onkelinx, et al.^54^ to estimate the yearly population totals of the bunkers (with standard errors) in the presence of missing data. Onkelinx, et al.^54^ compared this method with other imputation methods using a dataset of waterbird counts in terms of bias and variance, and concluded that their multiple imputation method performs better than other existing imputation methods. To carry out the multiple imputation we first modelled the complete available dataset with a generalized linear model with a negative binomial distribution. To estimate values for the missing data points, we randomly drew all model parameters (intercept, regression parameters and their standard deviations) from their respective distributions and then randomly drew imputation values from a negative binomial distribution with the drawn model parameters and the number of bats per bunker as independent variables. This step was repeated 1000 times to create 1000 imputed datasets. To model the barbastelle numbers against a dependent variable, modelling was carried out on all 1000 imputed datasets with a negative binomial regression model. Finally we calculated the overall regression parameters $\overline{\beta }_{i}$ and their standard errors $\overline{\sigma }_{i}$ following Rubin’s rules^55^:

$$\begin{aligned} \overline{\beta }_{i} & = \frac{1}{N}\mathop \sum \limits_{n = 1}^{N} \hat{\beta } \\ \overline{\sigma }_{i}^{2} & = \frac{1}{N}\mathop \sum \limits_{n = 1}^{N} \hat{\sigma }^{2} + \frac{N + 1}{N}\mathop \sum \limits_{n = 1}^{N} \\ \end{aligned}$$

where $N$ is number of imputed datasets (in our case N = 1000), $\overline{\beta }_{i}$ is the overall regression parameter i, $\hat{\beta }$ is the estimated regression parameter i from imputed dataset n, $\overline{\sigma }_{i}$ is the overall standard deviation for regression parameter i and $\hat{\sigma }$ is the estimated standard deviation for regression parameter i from imputed dataset n. The overall AIC value was calculated as the mean of the N AIC values of the modeling of the N imputed datasets.

The average of $\hat{\beta }$ over all N imputed datasets is an unbiased estimate of the true index $\beta_{i}$. The squared standard error $\overline{\sigma }_{i}^{2}$ of this average, $\overline{\beta }_{i}$, is the sum of two components^56^. The first is the average of the squared standard errors $\hat{\sigma }^{2}$ of the individual $\hat{\beta }$ over the N imputated datasets and is a measure of within-imputation variability. The second is the variance of $\hat{\beta }$ among the imputed datasets, i.e. the between-imputation variability, multiplied with a correction factor $\left( {L + 1} \right)/L$. This component will be large when the imputation step is highly variable.

Trends in barbastelle numbers were modelled with generalised linear models and generalised additive models with a negative binomial distribution and a log link. All models were compared with an intercept only null model without a trend term. Model comparison was based on AIC values, with models with an AIC at least two units lower than the null model being considered significantly better. AIC of a model on the imputed dataset was calculated as the average AIC of the modelling on all 1000 imputed datasets. Pearsons’ correlation was used to assess whether the trends in bat numbers seen in the underground and in the bunkers were correlated.

#### Software

All data were analysed with R version 3.6.1 R^57^. Generalized additive models (GAM) were fitted with the gam-function of the mgcv-package version 1.8–28^58^. Generalised linear models (GLM) with the glm.nb function of the MASS package version 7.3–51.4^59^.

## Results

### Temperatures inside the hibernacula

**Figure 1:**
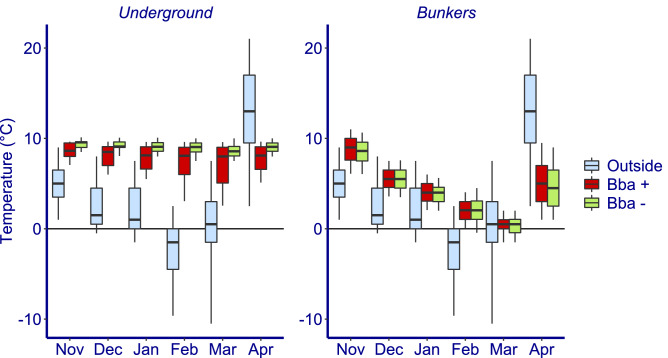
Temperatures (winter 2017/2018) of *Barbastella barbastellus* hibernation sites. The boxplots represent temperatures measured outside, in the bunker rooms and the subsections of the underground corridor system with *B. barbastellus* (Bba +) and without *B. barbastellus* (Bba −). Centre line, median; box limits, 25–75% quantiles; whiskers, 0.05–0.95% quantiles. Number of loggers (rooms/subsections) are respectively: Underground Bba + 48, Underground Bba- 52, Bunkers Bba + 16, Bunkers Bba − 39.

**Figure 2:**
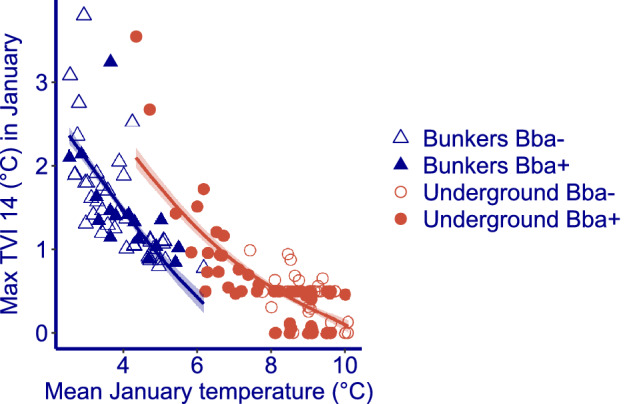
Maximum Thermal Variability Index in January against Mean January temperature for the bunkers and for the underground system. Bba + are subsections/rooms where *Barbastella barbastellus* was found during the mid January census of 2018. Bba- are subsections/rooms where *Barbastella barbastellus* was absent during the mid January census of 2018. Trend line = mean ± SE.

Temperatures in both the underground system and the bunkers were well above the outside temperatures apart from in the bunkers at the end of the winter in March (Fig. 1). In the underground system, hibernating *B. barbastellus* bats occupy the coldest sections, but are also found in a number of the warmer sections. During the course of winter, temperatures in the underground sections only decrease slightly because the system is well buffered. On average the sections containing *B. barbastellus* get slightly colder because they are situated closer to the entrances. However, part of the population also uses the warmer parts of the system which is illustrated in Fig. 1 by the increasing quantile range as winter progresses. In contrast to the underground system, temperatures in the surface located bunkers decrease strongly until they reached freezing conditions in March (Fig. 1). The seasonal variation, taken over a whole winter season, of the temperatures is much higher in the bunkers than in the underground system. For the above ground bunkers, we found no difference in temperature between the sites where *B. barbastellus* was found and where *B. barbastellus* was absent. In November, there is overlap in temperatures that are available between the coldest sections of the underground system and the bunkers (Fig. 1). This overlap gradually disappeared to end of the winter in March. However, in January there still was an overlap in mean January temperature between the coldest sections of the underground system and the bunkers (Fig. 2).

In the winter 2017–2018, when inside temperatures were monitored, B. *barbastellus* was found in hibernation sites with mean January temperatures ranging between 2.5 and 10 °C. In the deep underground system, 53% of the bats (354 individuals) were found at sites with a mean January temperature above 6.5 °C, 36% (238 individuals) at sites with a mean January temperature above 8 °C. In the bunkers all sites used by hibernating *B. barbastellus* had a mean January temperature below 5.5 °C.

Short-term variability, as depicted by the Thermal Variability Index, is higher at sites with a low mean January temperature (Fig. 2). A regression model of the TVI_14_ against the mean January temperature reveals a lower short-term variability as the mean January temperature is higher. This pattern is found in the underground system as well as in the above ground bunkers. However, the regression line for the above ground bunkers lies well below the regression line for the underground system. In the colder sections of the underground system, thermal variability was higher than in the bunker rooms with comparable mean temperatures. The difference between bunkers and underground is highly significant (model with object type: AIC = 143; model without object type: AIC = 171). Thus, the relationship between long-term and short-term variability is system dependent, and microclimates with a different short-term thermal variability for the same inside mid-winter mean temperature can occur if different types of systems are available.

### Long term *Barbastella barbastellus* trends

**Figure 3:**
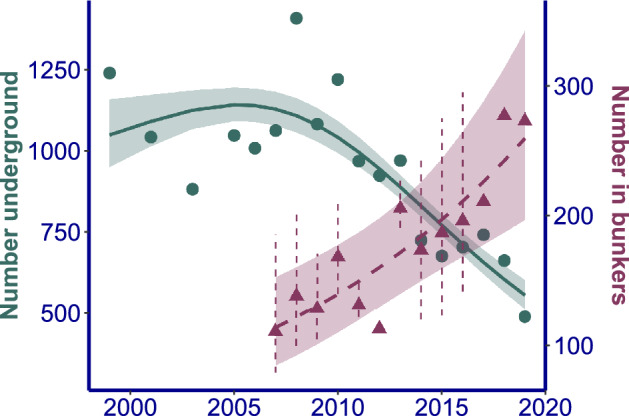
Population trend of *Barbastella barbastellus* in the underground system and the bunkers. Underground (●): yearly total count of the underground corridor system. Bunkers (▲): yearly total count in bunkers after imputation of missing data. Vertical dashed lines represent the 95% confidence interval for partly imputed values. Trend line = mean ± SE for the best fitting model: GAM for the underground and GLM for the bunkers.

Numbers of *B. barbastellus* in the underground system varied between 900 and 1400 individuals during the first decade, but significantly decreased thereafter to less than 500 by 2019. The trend was highly non-linear (AIC GAM = 228, AIC GLM = 239, AIC null model = 251; Fig. 3). At the same time, there was a significant, exponential increase in numbers of *B. barbastellus* in the bunkers (AIC GAM = 133, AIC GLM = 132, AIC null model = 145). A GAM model did not perform better than a GLM model, possibly reflecting the relatively short duration of the time series (13 years). The increasing trend in the bunkers is significantly correlated with the decreasing trend in the underground system (Pearson r^2^ = 0.48, df = 11, *p* = 0.008). The decrease in the last decade in the underground system corresponds with a yearly population decline of 8.85%, the increase in the bunkers in the same period with a yearly population increase of 8.93%.

### Temperature trends

**Figure 4:**
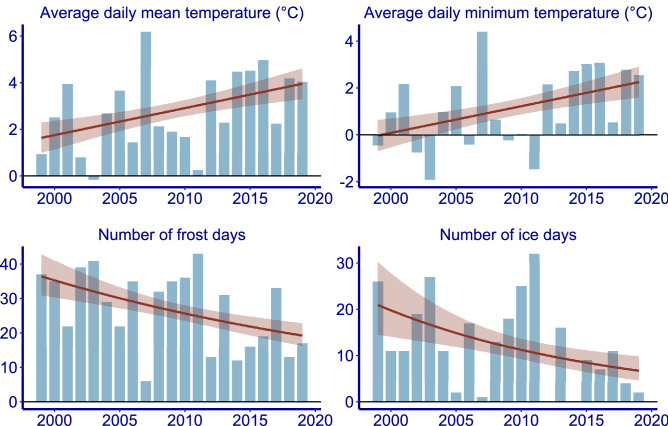
Trends in climatic parameters between 1 November and 15 January over the study period. Red: model fit line with a GLM model + /− standard error.

**Figure 5:**
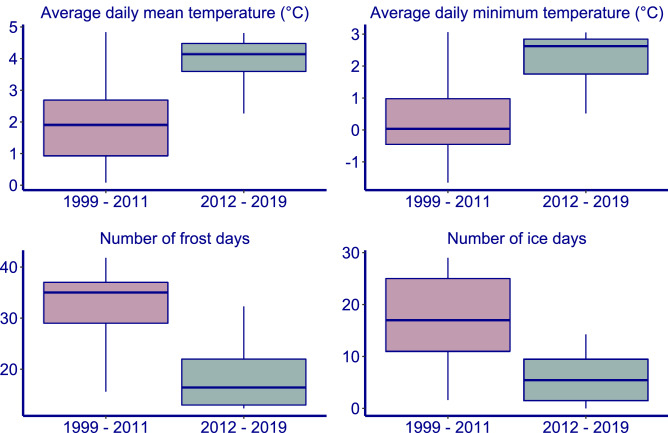
Climatic parameters for the period 1999–2011 and 2012–2019. Centre line, median; box limits, 25–75% quantiles; whiskers, 0.05–0.95% quantiles.

There was a significant increase in average daily mean and minimum temperature, and a significant decrease in number of frost days over the study period (Table 1; Fig. 4). From 2012 onwards weather conditions were characterized by a consecutive period of warmer winters. Warm winters also occurred in the period before, but not as a long consecutive series. Temperatures from 2012 onwards were clearly higher than before, and the number of frost and ice days was clearly lower than before (Fig. 5).

**Table 1:** AIC values of the trend modelling in winter parameters.

Parameter	Trend estimate	Standard error	AIC	AIC Null model
Average daily mean temperature	0.115	0.056	**82.30**	84.47
Average daily minimum temperature	0.114	0.057	**82.46**	84.49
Number of frost days	-0.0319	0.0144	**161.29**	164.08
Number of ice days	-0.0568	0.0320	150.72	151.47

Null model is without trend. Trends with an AIC at least two lower than the null model are marked in bold. For Number of frost days and Number of ice days a log link was used.

## Discussion

### Thermal conditions in hibernacula

*Barbastella barbastellus* used hibernation sites with a broader range of temperatures than expected given findings from previous studies^13,42^. We predicted that *Barbastella barbastellus* predominantly uses the colder available microsites, but 53% of the population in the deep underground system used sites with a mean January temperature above 6.5 °C, in contradiction to temperature ranges reported previously^13^ and to the expectation for a psychrophilic species. This indicates that, in line with other bat species, there is also considerable individual variation in hibernation requirements in this species. Boyles, et al.^32^ similarly found that hibernating Indiana bats (*Myotis sodalis*) select mid-winter microsites with a wider range of environmental conditions than is often stated. Our data on *B. barbastellus* confirm their conclusion that there is no “optimal” temperature for bat hibernation. However, when we merge the numbers hibernating in the bunkers and the underground system, about 2/3 of the population hibernates at colder temperatures.

Our data show that bunkers offer generally colder environments than the underground system, in accordance with our prediction. There is, however, overlap in temperatures between sites used by *B. barbastellus* in both systems. In 2017–2018 the microclimate in the bunkers only reached freezing conditions at the end of the winter. In colder winters these conditions will be reached earlier in the season, making the bunkers less suitable for hibernation. In warmer winters however, they are suitable, and extent the range of available microclimates for hibernation of *B. barbastellus*. Additionally, for comparable cold mean temperatures, somewhat surprisingly, bunkers offer a more stable environment than the underground system as shown by the Thermal Variability Index. In the above ground bunkers, the seasonal variability is higher, giving rise to lower mid-winter temperatures. For a given mid-winter temperature, the short-term variability in the bunkers is lower than in the underground system. Therefore, our prediction that thermal variability is higher in the above ground bunkers than in the underground system is only partly true. It holds for seasonal variability, but not for short-term variability. When searching for cold hibernation conditions, *B. barbastellus* may find a more stable environment in the bunkers than in the underground system. This can be explained by the different physical nature of both types of hibernation structures.

In a deep underground system, conductive temperature transport through the walls and the ground cover will be negligible. Deep parts of the system therefore will have a constant temperature throughout the year, as is found in the Nietoperek system (Fig. 1). On the other hand, conduction through the walls will not be negligible in a bunker, leading to a general decrease of the wall temperatures over the winter season (Fig. 1). However, in, a deep underground system with multiple entries*,* like the underground system of Nietoperek, temperature will also be determined by air flow, which in its turn is mainly determined by thermal convection^60^. The principle of thermal convection in a multiple entry underground system is that air enters through a lower entry and escapes (rises) through an upper entrance when it is warmer than the outside air. Therefore, in winter, cold air from the outside entering through a lower entrance, heats up because of heat exchange with the walls during its passage through the system and finally reaches an equilibrium when the air has the same temperature as the system walls. The heated air then leaves the system through the upper entrances. Close to the entrance of the air flow, temperatures are not yet equilibrated and will fluctuate, the rate of which depending on the speed of the air flow. The chimney effect can create relatively high speeds of air flow^28^, and therefore fluctuations can be considerable. In an above ground bunker system there is no chimney effect, and air flow is determined by wind. Because of the lack of a chimney effect and the smaller sized corridors with many turns (Supplementary Figure S.2), air flow will be much lower in a bunker than in the underground system. Short term fluctuations therefore will be less pronounced in bunkers, except for rooms with a direct opening to the outside. Therefore, bunkers can offer a more stable environment at lower mean temperatures. Moreover, the bunkers offer a great variety of cracks and fissures in which the bats can hide. This way, bats can further regulate their hibernation microclimate, hiding away from the influence of the air flow. Indeed, during the 2018 census 74% of *B. barbastellus* found in the bunkers were hidden in crevices*,* whereas in the underground system fewer crevices are available and nearly all *B. barbastellus* hibernate open against the walls. Our findings are similar to other studies in similar small systems where *B. barbastellus* was often found hibernating in crevices^44,61^.

### Trends in hibernation microsite use

Winter severity has declined during the last two decades, with higher mean and minimum temperatures and a lower number of frost and ice days. This is especially true from 2012 onwards where weather conditions have been characterized by a long consecutive period of warmer winters.

The numbers of *B. barbastellus* in the underground system of the Natura 2000 site "Nietoperek" declined during this period of milder winters. Numbers of *B. barbastellus* in the same underground system for the preceding period from 1985 to 1996 were reported by Lesinski, et al.^61^, during which time the numbers of *B. barbastellus* increased until 1990, and thereafter stabilised to within the range we see in the first decade of our study period. This suggests that the observed decline within the last decade is likely to be a recent phenomenon. At the same time, the numbers of *B. barbastellus* in the above ground bunkers around the underground system increased with both time series being negatively correlated. In the first decade mild winters also occurred, but not in consecutive series as was observed from 2012 onwards. Therefore, a single mild winter is not sufficient to observe a reaction in the population of *B. barbastellus* in Nietoperek, but rather a consecutive series of milder winters is required.

Bats, including *B. barbastellus*, often show site fidelity to their hibernation places^15,16,29,62^. Daan^29^ found that even when bats switch places during hibernation, they still only use a select number of places each year. Therefore, if the availability of suitable hibernation sites is expanded, part of the population might switch hibernation locations, but part of the population will stay. This may explain our observation that numbers of *B. barbastallus* only decreased gradually in the Nietoperek underground system. In the Nietoperek underground system the yearly population decline was 8.85% and the yearly population increase in the bunkers was 8.93%. Dietz, et al.^47^ state an expected average life span for *B. barbastellus* ranging from 5.5 to 10 years, corresponding to an annual mortality of 9.5 to 16.6%^47^. There is a striking correspondence between the lower value of this range and the rate of change in the underground system and in the bunkers. Together with the aspect of site fidelity, it may suggest that juveniles are predominantly responsible for the habitat shift by selecting the colder bunker sites that now belong to the available range of habitats given the milder winter temperatures. This, however, needs to be confirmed by further research.

Our prediction that in warm winters the underground system becomes too warm for hibernating *B. barbastellus,* forcing them to search for alternative hibernation sites, is not supported by the temperature data. Rather, in mild winters the range of suitable hibernation habitats for *B. barbastellus* is extended, and potentially even better environments become available. Since only part of the population will select these new opportunities, a gradual decline in the number of hibernating bats in the underground will only become detectable after a prolonged series of warm winters, such as we are currently experiencing.

### Seasonal variability and short-term variability in temperature

Microsite selection in relation to thermal variability has been addressed in several studies^28,32,62–64^. However, the distinction between long-term variability and short-term variability in temperature has never been made, except by Boyles, et al.^32^. Most studies assume that a higher seasonal variability, giving rise to lower mid-winter temperatures, implies a higher short-term variability. Our study however shows that this distinction can be important when examining microsite selection by hibernating bats. This is especially true when systems with different physical characteristics are available to the bats. In our study region bats can choose between a multiple entry underground system and above ground bunkers. In regions with more natural caves, a variation in types of caves can be available in which a different relationship between seasonal variability and short-term variability exists, since the air flow patterns will differ from cave to cave. For instance, single entry caves will behave completely different from multiple entry caves.

We investigated short-term variability in temperature in relation to microhabitat selection for a psychrophilic species, *Barbastella barbastellus*. We are convinced that short-term variability in temperature can also be important for other species. Martinkova, et al.^63^ showed that part of the thermophilic species *Myotis myotis* moves to colder parts near the entrance of a cave in the Czech Republic towards the end of the winter season. *M. myotis* in the colder parts then forms larger clusters. Barbastelle bats in our study sometimes hibernate in clusters, but more than half of the population hibernates solitary, even at low temperatures. Similar movements at the end of the season were reported by Daan and Wichers^28^ for *Myotis Myotis*, *Myotis dasycneme*, *Myotis daubentonii* and *Myotis mystacinus* in the Netherlands.

When bats choose colder sites at the end of the winter to reduce their energy expenditure, a low short-term variability can be beneficial. However, they also could use variation in temperature as a signal that conditions outside the cave are such that successful foraging might be possible or that it is time to migrate^32^. In that case, a high short-term variability can be advantageous. Therefore, making the distinction between seasonal variability and short-term variability in temperature can be a key to further understanding of microsite selection by hibernating bats.

## Conservation implications

Under climate change, winter conditions are predicted to get milder, with an increase in temperatures and a decrease in the number of frost and ice days^35–37^. For bats hibernating in deep underground systems, a gradual habitat shift such as we found here will be the first step in adapting to the new conditions. Microclimatic heterogeneity can buffer species against regional extirpations as has been shown for plants and insects^65^. Depending on the availability of small scale objects, the population may find even better conditions for hibernation, since this type of objects can offer more stable conditions at low temperatures. However, this situation is expected to be temporary, because after some time these objects will also become too warm due to rising winter temperatures. Bats can then shift to hibernating in trees, which means they stay in their summer habitat, but finally the population will become locally extinct. However, our analysis shows that the presence of small scale objects in the neighbourhood of an underground system, and by extension old grown forests with tree holes and loose bark, can temporarily mitigate the first stages of climate change. It also emphasises the high conservation value of such locations. Since hibernating bats are sensitive to human disturbance^66,67^, small bunkers should be as well protected as the underground system, and safeguarded from human visitors during the hibernation season.

## Supplementary Information

Supplementary Figures.
