# Supplementary material for: Temperature driven hibernation site use in the Western barbastelle Barbastella barbastellus (Schreber, 1774)
Source: Sci Rep. 2021 Jan 14;11:1464. doi: 10.1038/s41598-020-80720-4 (PMC7809113; doi:10.1038/s41598-020-80720-4)
Supplement: Supplementary file 1 — Supplementary Figures. [file 41598_2020_80720_MOESM1_ESM.pdf]

Temperature driven hibernation site use in the Western barbastelle *Barbastella barbastellus* (Schreber, 1774).

Luc De Bruyn [1,2,+], Ralf Gyselings [1,+], Lucinda Kirkpatrick [2], Alek Rachwald [3], Grzegorz Apoznański [4] and Tomasz Kokurewicz [4,*]

[1] Research Institute for Nature and Forest (INBO), Brussels, Belgium

[2] Evolutionary Ecology Group, University of Antwerp, Antwerp, Belgium

[3] Forest Research Institute, Forest Ecology Department, Sękocin Stary, Poland

[4] Wrocław University of Environmental and Life Sciences, Institute of Environmental Biology, Department of Vertebrate Ecology and Paleontology, Wrocław, Poland

[+] Shared first authorship

[*] Corresponding author: tomasz.kokurewicz@upwr.edu.pl

Supplementary material

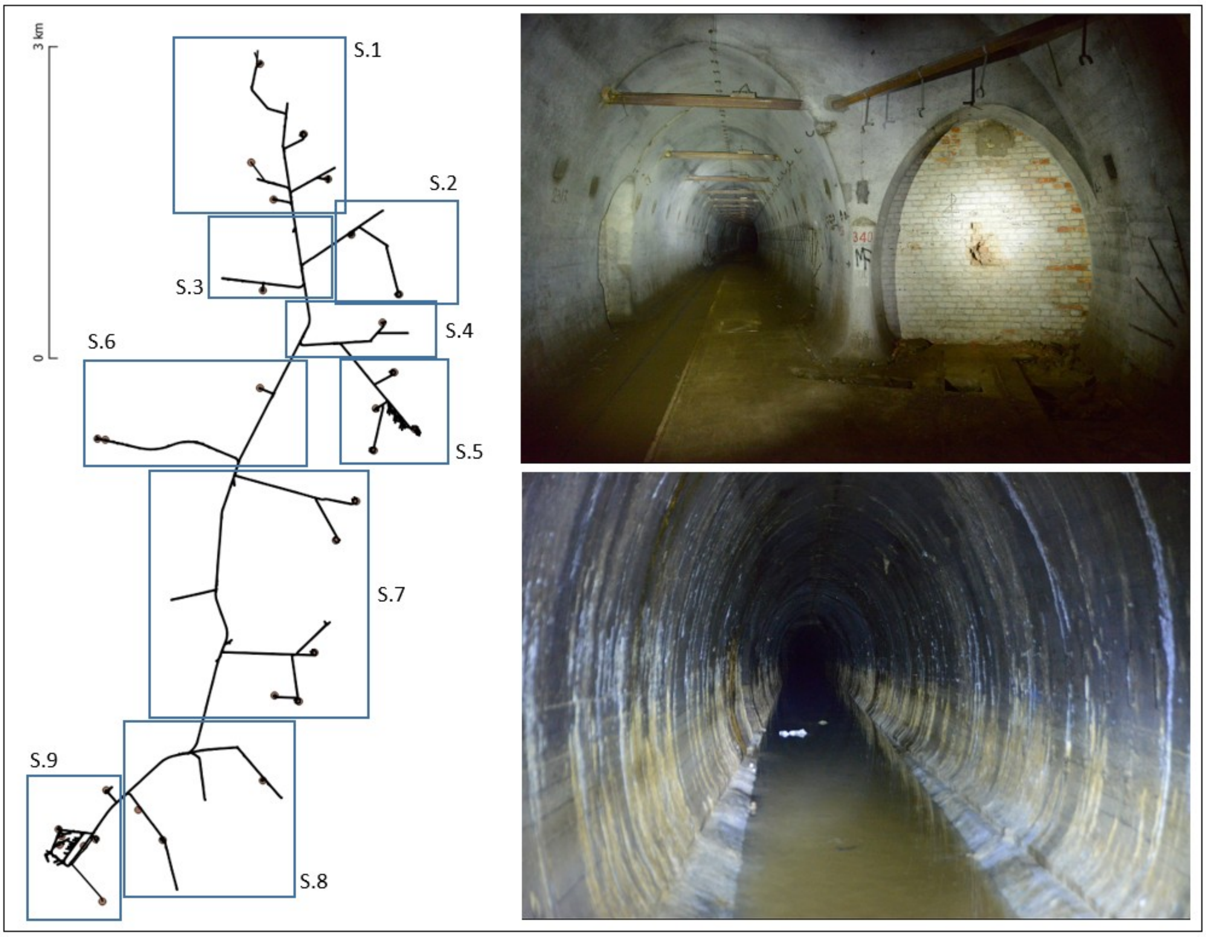

Figure S.1: Underground corridor system. Left: ground plan with nine sections. Right: Two examples of corridors: top: main corridor, bottom: a side corridor.

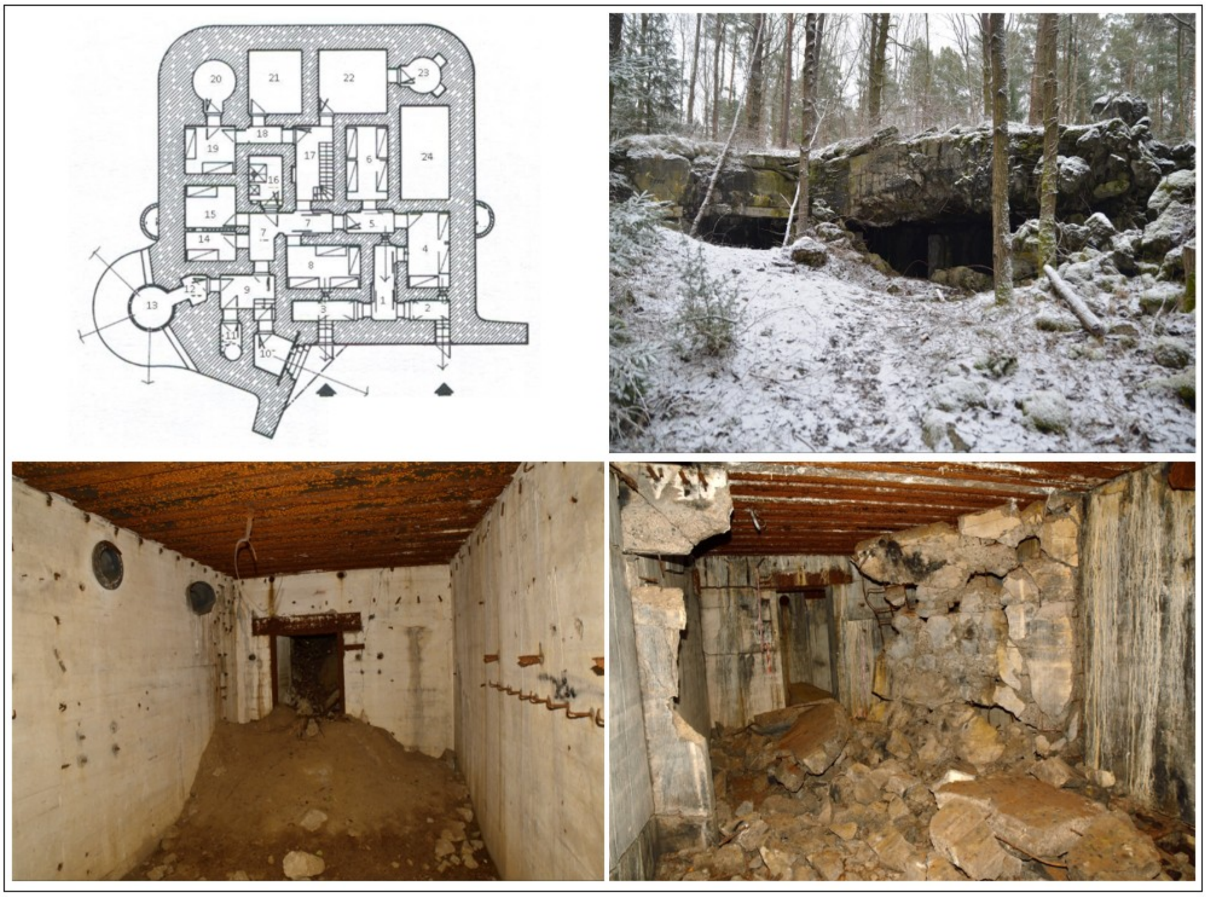

Figure S.2: Aboveground bunkers. Top left: Ground plan of one of the bunkers, after Jurga & Kędryna [68]. Top right: A bunker entrance. Bottom: Two examples of bunker rooms.
